# Automated reporting of safety bundles: streamlining the performance improvement process

**DOI:** 10.1186/cc9899

**Published:** 2011-03-11

**Authors:** W McGee, T Higgins, J Echols, H Nelson, M Tidswell

**Affiliations:** 1 Baystate Medical Center, Springfield, MA, USA

## Introduction

Safety checklists, long used in aviation, have migrated to the critical care setting in an effort to reduce complications and improve patient outcomes. We developed an automated system to provide real-time feedback to the healthcare team on safety bundle compliance in the ICU.

## Methods

A program was written in Cerner Command Language to automatically search data within the EMR for the most recent values of the following data: ventilation mode, respiratory rate, tidal volume, ideal body weight, administration of any sedative infusion, analgesic infusion, neuromuscular blocking agent, stress ulcer prophylaxis, DVT prophylaxis and regular diet, enteral nutrition, or total parenteral nutrition. Nursing documents oral decontamination, head-of-bed elevation, and whether a sedation vacation was conducted. A customized document was created to capture any patient/family/proxy discussions about end-of-life issues. High and low glucose values and the percentage of all glucose values within the range of 60 to 180 mg/dl are also reported. A summary score was calculated by subtracting (from a baseline of 9) one point each for inappropriate tidal volume, failure to do a weaning trial, lack of oral care, head-of-bed elevation and stress prophylaxis, no sedation vacation and absent DVT prophylaxis, absent nutritional support or glucose values out of range. A perfect score is 9; lower scores indicate an opportunity for improvement. Clinicians access the report from within the hospital's EMR and can view it on a portable device (iPad) or print it to carry on rounds.

## Results

A partial report with data for two patients is shown in Table 1.

**Table 1:** ICU safety bundle report

Loc	Vent	f/Vt	Vt/IBW	Stress	NM block	Oral	HOB	DVT	24-hour glucose	% glu	Pain	Sed	Sed vac	Nutr	Score
4202	AC	0	6	Y	N	Y	Y	Y	105.216	67	Y	N	Y	R	6
4203	PSV	15	Y	N	N	Y	Y	Y	NA	0	Y	N	Y	R	6

## Conclusions

Automatic reporting of compliance with patient safety protocols is a useful performance improvement tool that further identified areas for improvement. Future study should assess the impact of this tool on actual compliance with patient safety goals.

